# Letter to the editor: Sampling bias should be minimised when analysing influenza transmission zones involving very large countries

**DOI:** 10.2807/1560-7917.ES.2017.22.40.17-00664

**Published:** 2017-10-05

**Authors:** Gee Yen Shin, Rohini Manuel

**Affiliations:** 1 Department of Microbiology, London North West Healthcare NHS Trust, Northwick Park Hospital, Harrow, United Kingdom; 2 Department of Medical Microbiology, Epsom and St Helier University Hospitals, NHS Trust, St Helier Hospital, Carshalton, United Kingdom

**To the editor:** We read Caini et al.’s detailed analysis of the World Health Organisation (WHO) European region influenza transmission zones (ITZ) and their own ITZ proposal with interest [1]. The authors state that the WHO definition of ITZs is:

“…Influenza Transmission Zones are geographical groups of countries, areas or territories with similar influenza transmission patterns.”

Their map of the WHO European region ITZ (Figure 1) and the graphical representations of peak influenza epidemics by longitude (Figure 2) and latitude (Figure 3) raise questions around the completeness of the representation and analysis of influenza activity in Russia, given its large land area.

The authors briefly allude to our central concern of this kind of analysis in their discussion of their paper’s limitations: “

…it would be desirable to have regional data for the Russian Federation…”.

We agree with this, but feel that this point is important and deserves more scrutiny.

For their spatiotemporal analysis, the authors used the national influenza centres (NIC) of each country in the European region. For countries with more than one NIC, they selected the NIC in the largest city. While this approach is logical, in the case of very large countries such as Russia, we perceive potential limitations with this strategy.

Russia is the largest country in the world, with a land area of 16,377,742 km^2^. It has 11 time zones. The land area of Russia is almost four times the combined land area of the whole of the European Union at 4,479,968 km^2^ [2]. We can understand that choosing one data point, i.e. the NIC for each country, eases spatiotemporal analysis. However, we are sceptical that looking at influenza activity in only one city, i.e. Moscow, gives an accurate picture of what is happening in that vast country.

Similarly, we have reservations about the validity of plotting one point (Moscow) to represent national influenza transmission trends by latitude and especially longitude given Russia’s size. It seems unlikely that the influenza activity is uniform or even close to uniform across such a large country with notably varied geography, climate and demography. We understand that Russia is home to almost 200 ethnic groups [2]. For these reasons, we would hypothesise a complex spatiotemporal pattern of influenza transmission exists within Russia (and other very large countries).

Therefore, we are concerned that relying on one NIC in very large countries could result in sampling bias and may not accurately represent influenza activity in the whole country.

It would be interesting to see a similar analysis of influenza transmission within Russia looking at other major cities such as Novosibirsk, Yekaterinburg and Omsk, all cities with a population greater than a million and somewhat distant from Moscow. We realise there are no NICs in these cities, but we assume it would be possible to gather influenza activity data from these relatively large cities.

Given the vastness of Russia, we would not be surprised if a more detailed analysis of influenza transmission there suggested Russia could be an ITZ on its own. Indeed, for all the above reasons, Russia could potentially encompass more than one ITZ.

We discern no obvious correlation between a country’s size and how many NIC’s it has. Russia has two NICs, Moscow and St Petersburg. We note that the United Kingdom, a relatively small country (land area 241,930 km^2^), has four NICs [3]. We would support the establishment of more NICs in very large countries such as Russia, Canada and China, resources and political will permitting. This would improve national influenza surveillance schemes, and strengthen global influenza surveillance.
